# The Cancer Moonshot Immuno-Oncology Translational Network at 5: accelerating cancer immunotherapies

**DOI:** 10.1093/jnci/djad151

**Published:** 2023-08-12

**Authors:** Ananth Annapragada, Andrew G Sikora, Himangi Marathe, Song Liu, Michael Demetriou, Lawrence Fong, Jinming Gao, Donald Kufe, Zachary S Morris, Eduardo Vilar, Elad Sharon, Alan Hutson, Kunle Odunsi

**Affiliations:** Edward B. Singleton Department of Radiology, Texas Children’s Hospital and Baylor College of Medicine, Houston, TX, USA; Department of Head and Neck Surgery, Division of Surgery, The University of Texas MD Anderson Cancer Center, Houston, TX, USA; Department of Biostatistics and Bioinformatics, Roswell Park Comprehensive Cancer Center, Buffalo, NY, USA; Department of Biostatistics and Bioinformatics, Roswell Park Comprehensive Cancer Center, Buffalo, NY, USA; Department of Neurology, University of California, Irvine, Irvine, CA, USA; Department of Microbiology and Molecular Genetics, University of California, Irvine, Irvine, CA, USA; Department Hematology and Oncology, Department of Medicine, University of California San Francisco, San Francisco, CA, USA; Parker Institute of Cancer Immunotherapy, San Francisco, CA, USA; Department of Pharmacology, Harold C. Simmons Comprehensive Cancer Center, University of Texas Southwestern Medical Center, Dallas, TX, USA; Department of Otolaryngology, Harold C. Simmons Comprehensive Cancer Center, University of Texas Southwestern Medical Center, Dallas, TX, USA; Department of Biomedical Engineering, Harold C. Simmons Comprehensive Cancer Center, University of Texas Southwestern Medical Center, Dallas, TX, USA; Department of Medical Oncology, Dana-Farber Cancer Institute, Harvard Medical School, Boston, MA, USA; Department of Human Oncology, University of Wisconsin-Madison, Madison, WI, USA; Department of Gastrointestinal Medical Oncology, The University of Texas MD Anderson Cancer Center, Houston, TX, USA; Department of Clinical Cancer Prevention, The University of Texas MD Anderson Cancer Center, Houston, TX, USA; Department of Medical Oncology, Dana-Farber Cancer Institute, Harvard Medical School, Boston, MA, USA; Department of Biostatistics and Bioinformatics, Roswell Park Comprehensive Cancer Center, Buffalo, NY, USA; University of Chicago Medicine Comprehensive Cancer Center, Chicago, IL, USA; Department of Obstetrics and Gynecology, The University of Chicago, Chicago, IL, USA

## Abstract

The Immuno-Oncology Translational Network (IOTN) was established in 2018 as part of the Cancer Moonshot. In 2022, President Joe Biden set new goals to reduce the cancer death rate by half within 25 years and improve the lives of people with cancer and cancer survivors. The IOTN is focused on accelerating translation of cancer immunology research, from bench to bedside, and improving immunotherapy outcomes across a wide array of cancers in the adult population. The unique structure and team science approach of the IOTN is designed to accelerate discovery and evaluation of novel immune-based therapeutic and prevention strategies. In this article, we describe IOTN progress to date, including new initiatives and the development of a robust set of resources to advance cancer immunology research. We summarize new insights by IOTN researchers, some of which are ripe for translation for several types of cancers. Looking to the future, we identify barriers to the translation of immuno-oncology concepts into clinical trials and key areas for action and improvements that are suitable for high-yield investments. Based on these experiences, we recommend novel National Institutes of Health funding mechanisms and development of new resources to address these barriers.

In his final State of the Union address in January 2016, former President Barack Obama tasked then–Vice President Joe Biden to head a new national effort called Cancer Moonshot to “end cancer as we know it.” Cancer Moonshot has a specific aim of accelerating cancer research to make more therapies available to more patients and improve cancer prevention and early detection. Following this recommendation, in 2018 the National Cancer Institute (NCI) established the Immuno-Oncology Translational Network (IOTN) to serve as the premier collaborative network and resource focused on immunotherapy and immunoprevention. In 2022, President Biden set new goals of “achieving a decade’s worth of progress in 5 years” and “to reduce the cancer death rate by half within 25 years” (1). Accomplishing these new goals is a major challenge that will require tremendous innovations (2-4). The IOTN is poised to play a key role and proposes some avenues and innovations in that context. This article describes the IOTN’s progress and findings over the first 5 years of the consortium and proposes new resources and approaches to overcome barriers to rapid translation of research findings.

The IOTN is focused on accelerating the translation of cancer immunology research from bench to bedside and improving immunotherapy outcomes across a wide array of cancers in adult populations (5,6). The overarching goals of the IOTN are 1) to develop robust cancer immunotherapies while defeating the immunosuppressive tumor microenvironment and 2) to develop vaccines and immunotherapies to prevent cancers (7). The IOTN has expanded from 14 grant awards in 2018 to 31 awards by 2020, forming an extensively collaborative network spanning 33 institutions (Figure 1). Two institutions are U24 resource centers. The remaining 29 centers (23 U01, 2 UG3, and 4 U54 centers) constitute 4 major themes: 1) immunotherapy, 2) immuno-engineering to improve immunotherapy, 3) mitigating immune-related adverse events, and 4) immunoprevention consistent with the goals of the president’s blue-ribbon panel (5,6).

**Figure 1.**
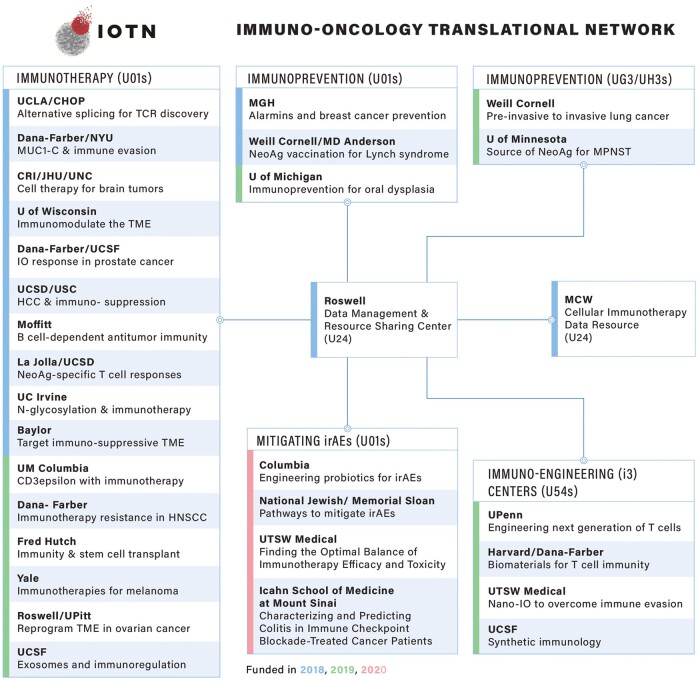
**IOTN network structure.** The current research themes and associated awardee institutes making up the IOTN. CHOP = Children’s Hospital of Philadelphia; CRI = Children’s National Research Institute; Fred Hutch = Fred Hutchinson Cancer Center; HCC = hepatocellular carcinoma; HNSCC = head and neck squamous cell carcinoma; IO = immuno-oncology; IOTN = Immuno-Oncology Translational Network; irAE = immune-related adverse event; JHU = Johns Hopkins University; MCW = Medical College of Wisconsin; MGH = Massachusetts General Hospital; MPNST = malignant peripheral nerve sheath tumor; MUC1-C: Mucin 1(MUC1) C-terminal subunit; Nano-IO = Nano-Immuno-Oncology; NeoAg = Neoantigen; NYU = New York University; Roswell = Roswell Park Comprehensive Cancer Center; TCR = T-cell receptor; TME = tumor microenvironment; U = University; UC = University of California; UCLA = University of California Los Angeles; UCSD = University of California San Diego; UCSF = University of California San Francisco; UM = University of Missouri; UNC = University of North Carolina; UPenn = University of Pennsylvania; UPitt = University of Pittsburgh; USC = University of Southern California; TSW = University of Texas Southwestern; U01s/UG3/UH3s = specific types of grants issued by the U.S. National Institutes of Health or the U.S. Department of Defense. This figure is an updated version of a figure previously published elsewhere (5).

The IOTN consists of a steering committee (31 principal investigators and the National Institutes of Health [NIH] Implementation Team); its working groups, including the Clinical Trials Task Force (CTTF); and 2 cross-network data-sharing resources (the Data Management and Resource Sharing Center [DMRC] and the Cellular Immunotherapy Data Resource [CIDR]). IOTN working groups focus on 5 previously identified cross-disciplinary scientific gap areas: 1) immune mechanism and recognition, 2) immuno-radiation therapy, 3) immunoprevention, 4) translational cellular therapy, and 5) bioinformatics and computational biology. Membership is cross-fertilized with allied Moonshot networks, including the Pediatric Immunotherapy Discovery and Development Network (PI-DDN), Cancer Immune Monitoring and Analysis Centers–Cancer Immunologic Data Commons (CIMAC-CIDC), and the Pre-Medical Cancer Immunotherapy Network for Canine Trials (PRECINCT) networks.

## IOTN progress to date

### Interactions of the IOTN with existing groups and organizations

The IOTN has taken a collaborative and integrative approach to accomplishing Cancer Moonshot’s goal of “achieving a decade’s worth of progress in 5 years.” Speakers from other NIH and NCI initiatives are routinely invited to present at IOTN steering committee and working group meetings to enhance awareness of preclinical and clinical translational resources and to foster research collaboration. The fall 2021 IOTN semiannual steering committee meeting included a joint immuno-oncology day, with participation from the Pancreatic Cancer Microenvironment Network, PI-DDN, PRECINCT, and the CIMAC-CIDC networks. Joint participation in working group meeting discussions and steering committee meetings helps the NCI stay abreast of the network’s progress, identify necessary resources, and develop future initiatives. The 2 resource centers—DMRC and CIDR—also provide both intranetwork and cross-network collaboration opportunities.

The DMRC has created the IOTN website (https://www.iotnmoonshot.org/en/resources/all-resources/). The Data Sharing Catalog lists datasets deposited into NIH-designated repositories and associated with IOTN awards, currently including 55 datasets obtained from more than 3500 human and murine studies. The DMRC, in conjunction with the IOTN Immune Mechanism and the IOTN Translational Cellular Therapy working groups, created the Model Sharing Catalog, which inventories relevant immunocompetent preclinical models published by the IOTN. As of February 2023, the IOTN has contributed 11 preclinical models ranging from genetically engineered mouse models to xenografts and organoid models representing 9 cancer types. Additional resources include the Software Sharing Catalog and the Clinical Trials Catalog, combined IOTN/Drug Resistance and Sensitivity Network Data Sharing Catalog and Model Sharing Catalog databases created in collaboration with the Drug Resistance and Sensitivity Network. Finally, all IOTN publications are required to be immediately open access, thus supporting the network’s goal of disseminating research results in prompt and findable, accessible, interoperable, reusable ways (8). Other collaborative activities include co-hosting the Moonshot collaborative meeting in 2019, an NCI immunoprevention workshop in 2020, and a 9-webinar short course on computational immune-oncology with the Society for Immunotherapy in Cancer in 2021 and 2022.

### IOTN progress in preclinical research

The IOTN has been tremendously productive, with a total of 433 publications (as of June 9, 2023), of which 212 articles (49% of the total) were in journals with a 5-year impact factor greater than 10 and 74 articles (17.1% of the total) were published in top-tier journals with a 5-year impact factor greater than 30. IOTN research output includes exciting preclinical discoveries and potential therapies that could be moved into early-stage clinical trials. A few examples are highlighted here.

IOTN investigators are working to improve the limited efficacy of chimeric antigen receptor (CAR)-T cells in solid tumors. CAR-T cells entering solid tumors frequently enter an “exhausted” state triggered by chronic antigen stimulation and characterized by upregulation of inhibitory receptors. Chen et al. (9) and Seo et al. (10) showed that NR4A inhibition and BATF overexpression, respectively, may be promising strategies for overcoming T-cell exhaustion in the tumor microenvironment and improving CAR-T therapy efficacy. Other IOTN investigators using immuno-engineering approaches are working to reduce CAR-T cell “off-tumor” cross-reactivity with normal tissues that express low levels of target antigen. Hernandez-Lopez et al. (11) engineered a 2-step positive feedback circuit that allows T cells to discriminate targets based on a sigmoidal antigen-density threshold. A low-affinity synthetic Notch receptor for HER2 was designed to control expression of a high-affinity CAR for HER2, leading to a sigmoidal T-cell response (11). Moreover, using circuits that integrate recognition of multiple complementary antigens, Choe et al. (12) elegantly addressed challenges of specificity, heterogeneity, and persistence of CAR-T cells in preclinical models of glioblastoma. To endow adoptively transferred T cells with new functions that could overcome the need for conditioning chemotherapy, Kalbasi et al. (13) designed chimeric receptors that have an orthogonal interleukin 2 receptor extracellular domain fused with the intracellular domain of receptors for common γ-chain cytokines that demonstrated superior antitumor efficacy against hard-to-treat solid tumors.

IOTN investigators are also developing strategies to reverse immunosuppressive tumor microenvironments. For example, in ovarian cancer, Anadon et al. (14) demonstrated that the hallmarks of tumor recognition are primarily restricted to tissue-resident memory cells. Muthuswamy et al. (15) identified CXCR6 as a critical regulator of residency and persistence of memory CD8^+^ T-cell responses in the ovarian tumor microenvironment, supporting development of CXCR6/CXCL16-targeted therapeutic approaches to enhance antitumor tissue-resident memory retention within the tumor microenvironment. To gauge efficacy of tumor microenvironment–directed therapies, Devkota et al. (16) investigated a nano-radiomics approach (quantitative analysis of nanoparticle contrast–enhanced 3-dimensional images) for detection of tumor response to cellular immunotherapy. Animals bearing human myeloid-derived suppressor cell–containing solid tumor xenografts were treated with myeloid-derived suppressor cell–targeting human natural killer cells. Nano-radiomics revealed tumor immune contexture-based features capable of differentiating the impacts of natural killer cell immunotherapy on the tumor microenvironment, an approach that is potentially applicable to numerous solid tumors.

In head and neck and oral cancers, immune checkpoint inhibitor immunotherapy benefits only a small proportion of treated patients, and mechanisms of immunologic resistance must be identified to increase response rates. Wang et al. (17) described a HER3-PI3K-mammalian target of rapamycin signaling axis driving the immune-suppressive tumor microenvironment as well as a therapeutic vulnerability to dual-HER3/programmed cell death 1 protein blockade. A novel role for STING degradation by the pathogen recognition receptor NLRX1 was identified by Luo et al. (18) in human papillomavirus–related head and neck cancer, identifying NLRX1 as a potential therapeutic target. Other IOTN-supported publications provided new insights into immune-modulating properties of standard-of-care therapies such as chemotherapy and radiation therapy (RT) (19-22), a novel role for CD8 T-cell differentiation in oral cancer response to checkpoint inhibition (23), and a new mechanism of tumor microenvironment modulation and programmed cell death 1 protein resistance mediated by the human papillomavirus E5 oncogene (24). Collectively, these discoveries have the potential to increase the effectiveness of checkpoint inhibitor immunotherapy of head and neck cancer and other solid tumors.

Several exciting examples of potential combination therapy approaches have been developed by IOTN investigators. In the study by Pieper et al. (25), local antitumor effects of local RT combined with bempegaldesleukin, an investigational CD122-preferential interleukin 2 pathway agonist, resulted in a collaborative antitumor effect in all tumor models tested, and adding an immune checkpoint inhibitor to RT with bempegaldesleukin strengthened the antitumor response and cured most tumor-bearing mice. To capitalize on the immunogenic effects of RT, Patel et al. (26) studied targeted radionuclide therapy to deliver radiation semiselectively to tumors as an approach to enhance response to immune checkpoint inhibitors. The combination of a theranostic alkylphosphocholine analog, ^90^Y-NM600, with moderate-dose (12-Gy) external beam RT augmented responses to immune checkpoint inhibitors compared with a combination of immune checkpoint inhibitors with either targeted radionuclide therapy or external beam RT alone.

Single agents developed by IOTN investigators (Table 1) could potentially be combined with other therapeutic strategies, and the list demonstrates the phenomenal translational potential of the IOTN and its future impact on cancer immunotherapy strategies. For example, Conejo-Garcia’s team demonstrated that targeting BTN3A1 with antibodies against CD277 transform BTN3A1 from an immunosuppressive to an immunostimulatory molecule. This shift elicits a coordinated αβ and γδ T-cell–driven antitumor immunity that orchestrates cooperative killing of established tumors in an ovarian cancer model (27). This single agent can potentially be combined with other checkpoint inhibitors and alternative adoptive cellular therapy approaches [eg, anti-HER2 CAR-T cells (11) evaluated by other IOTN investigators] to enhance cancer immunotherapy responses. As another example, Ogunnaike et al. (28) demonstrated a delivery strategy involving fibrin gel that allows placement of CAR-T cells within the tumor resection cavity and promotes gradual release of loaded CAR-T cells for superior antitumor activity in glioblastoma.

**Table 1.** Potential clinical trial opportunities—single agents based on IOTN investigators' research

Agent	Category	Indication	Potential combination therapies	Reference
DR-18 (decoy resistant IL-18 binding protein)	Immune checkpoint	All solid tumors	—^a^	(36)
Bempegaldesleukin, a CD122-preferential IL-2 pathway agonist	Immune checkpoint	All solid tumors	Immune checkpoint blockade + radiation therapy	(25)
CD-19 reactive CAR-T cells with NR4A-triple knockout	Adoptive cell therapy	Solid tumors	NR4A inhibition + immune checkpoint inhibition	(9)
Anti-HER2 CAR-T (CD8-positive) cells	Adoptive T-cell therapy with chimeric receptors	HER2-expressing tumors	—^a^	(11)
Orthogonal IL-2 receptor extracellular domain fused with IL-9 receptor for intracellular domain for CAR-T cells	Adoptive cell therapy	Solid tumors	—^a^	(13)
Anti-BTN3A1 antibodies (clone CTX-2026)		Solid tumors	Adoptive cellular therapy and immune checkpoint	(27)
PD-L1 × CD3 bispecific antibody	Immune checkpoint	Solid tumors	Potentially combined with cellular therapy and BTN3A1 antibody, as well	(37)
SynNotch-CAR-T cells	Adoptive cell therapy	Glioblastoma	Novel delivery method, can be combined with immune checkpoint inhibitor and cellular therapy	(12)
B7-H3 CAR-T cells	Novel delivery method	Glioblastoma	—^a^	(28)
C/EBP homologous protein inhibition of CD8-positive T cells	Adoptive cell therapy	Ovarian, breast, and other solid tumors	—^a^	(38)
Two novel variant TGF-β receptors that couple the TGF-β–dominant negative receptor to intracellular signaling domains, mediating NK cell activation, 1) NKA: DNAX-activation protein 12 and 2) NKCT: synthetic Notch-like receptor	Adoptive cell therapy	Neuroblastoma	Combined with standard checkpoint inhibitors or novel checkpoint pathways described previously	(39)
CDN-Mn^2+^ particle (metalloimmunotherapy)	Vaccine	Solid tumors	—^a^	(40)
Nanoparticle STING agonist: a pH-sensitive polymer bearing a 7-member ring with a tertiary amine	Vaccine and immune activation	Solid tumors	Combined with checkpoint inhibitor therapy (anti–PD-1)	(41)
Combined theranostic alkylphosphocholine analog 90Y-NM600 with moderate-dose (12-Gy) external beam radiation therapy	Radioimmunotherapy	Virtually any tumor any location	—^a^	(26)
Four shared frameshift peptide neoantigens (Nacad [FSP-1], Maz [FSP-1], Senp6 [FSP-1], Xirp1 [FSP-1])	Immunoprevention vaccine approach	Patients with Lynch syndrome to prevent gastrointestinal malignancies	—^a^	(42)
Genetic transfer of T-cell receptor from neoantigen-specific T-cell clones into peripheral blood T cells was conducted to generate neoepitope-specific T cells	Vaccine	Ovarian and other solid tumors	—^a^	(43)
Neoantigen vaccine for identified tumor specific gene-gene fusion transcripts	Vaccine	Osteosarcomas and other solid tumors	—^a^	(44)
Multifunctional bacterial membrane-coated nanoparticle composed of an immune-activating PC7A/CpG polyplex core coated with bacterial membrane and imide groups + radiation	Radioimmunotherapy	Solid tumors	—^a^	(45)
CD8 T cells engineered with the CXCR6-CXCL16 axis	Vaccine and adoptive cellular therapy	Ovarian and other solid tumors	—^a^	(15)
Macroporous alginate gels loaded with granulocyte-macrophage colony-stimulating factor for concentrating dendritic cells, CpG oligonucleotides, and a doxorubicin-iRGD conjugate	Vaccine and chemoimmunotherapy	Breast and other solid tumors	—^a^	(46)
Glycan-dependent T-cell recruiters targeting tumor-associated carbohydrate antigens	Bispecific proteins and CAR-T cells	Solid and liquid tumors	Checkpoint inhibitors, multiple glycan-dependent T-cell recruiters	Unpublished (lab of Dr Demetriou)

a No value given because a single immunotherapy. CAR = chimeric antigen receptor; IL = interleukin; NK = natural killer; PD-1 = programmed cell death 1 protein; PD-L1 = programmed cell death 1 ligand 1; TGF-β = tyrosine kinases transforming growth factor β.

### The gap: difficulty translating preclinical discovery into actionable clinical trials

Among the Blue-Ribbon Panel recommendations was one specifically to set up a Clinical Trial Immunotherapy Network with the objective of constructing a nationwide infrastructure to facilitate immunotherapy trials. Despite major breakthroughs in understanding mechanisms of antitumor immunity and treatment resistance, translation to the clinic has lagged, in many cases because of unique challenges that apply specifically to immunotherapy. For example, gold-standard approaches used to measure the effectiveness and safety of traditional chemotherapy and antitumor drugs often do not apply to immuno-oncology agents. In addition, there is no clear dose-response relationship for such agents. Similarly, traditional clinical endpoints, such as overall survival and overall response rates, do not robustly apply to immuno-oncology agents because response kinetics may be delayed or confounded by differences in tumor response patterns. Off-target effects, which can often be beneficial in specific contexts, and the complexity of assessing immune-related adverse events make it challenging to perform standard toxicity evaluations.

To assist researchers in their design and clinical evaluation of immuno-oncologic agents, the IOTN committed to developing a resource to collect and make available robust patient data (disease specifics, demographics, treatment history) and details of therapeutic agent manufacturing to help identify markers of immune suppression and observed clinical outcomes to facilitate retrospective research (29,30). The IOTN established the CIDR in 2018 (31). The CIDR proved to be highly successful, providing a rich biorepository of more than 205 000 samples and supporting 250 clinical trials and ongoing studies as of 2022 (32).

### Establishment of the IOTN CTTF

During an “IOTN Future Directions” brainstorming session during the 2019 Cancer Moonshot Collaborative Meeting, IOTN investigators expressed a keen demand for new approaches and resources for clinical translation, given the particularly complex and time-consuming process of translating immunotherapies, and suggested establishment of networks connecting disparate elements to be in-sync for successful translation. At the following IOTN steering committee meeting in December 2019, a vibrant discussion ensued regarding establishment of an IOTN CTTF to fulfil the unmet need of rapid translation of preclinical discoveries into phase 1 trials. The kickoff meeting was held on January 15, 2020, and the CTTF was established under the leadership of Kunle Odunsi, MD, PhD, as chair and Andrew Sikora, MD, PhD, as co-chair. Supplementary Table 1 (available online) lists the members of the task force, which included IOTN investigators and key NCI officials.

**Figure 2.**
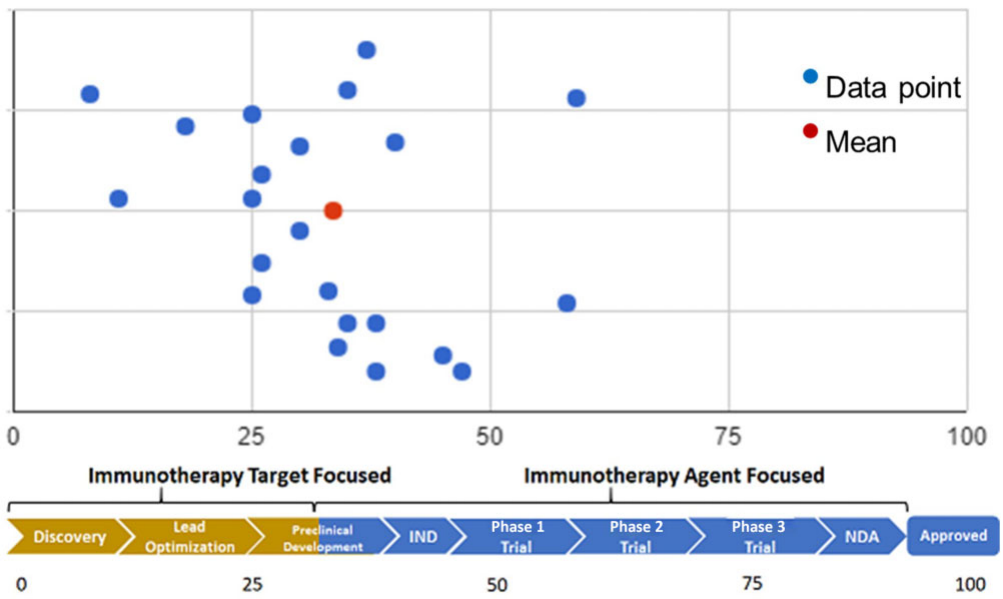
**Clinical Trials Task Force survey: response to the question of stage of target and/or agent on the clinical translation continuum.** Responses to the Clinical Trials Task Force survey conducted in 2021 of Immuno-Oncology Translational Network members. More than 50% of Immuno-Oncology Translational Network investigators had an immuno-oncology target or agent already in the pipeline for eventual translation into clinical trials, with the mean being at the preclinical development stage. IND = investigational new drug; NDA = new drug application.

The CTTF’s mission statement was “to engage IOTN investigators with academic, clinical, and industry partners; and to identify barriers to, and opportunities for, efficient translation of fundamental immunotherapy research into clinical testing.” Andrew Sikora, MD, PhD, and Ananth Annapragada, PhD, generated an initial Immuno-Oncology Road Map, cataloging both available resources and necessary steps for clinical translation of immuno-oncology concepts. The CTTF also carried out a survey of IOTN investigators to pinpoint stages of the journey, from preclinical development to clinical translation, where guidance was most needed. The survey results, summarized in Figure 2, provided a clear picture of the current status of the IOTN research projects, with an overwhelming 97% of respondents expressing interest in using clinical trial resources to accelerate translation. Most respondents indicated that they had either an immunotherapy target (50%) or an immunotherapy agent or a novel combination of existing agents (∼59%) in the pipeline for translation into clinical trials. Respondents indicated that most projects were at the intersection of early to mid-preclinical stage, working toward filing a US Food and Drug Administration (FDA) investigational new drug application. Focus areas requiring support for preclinical research included 1) production of drugs that meet good laboratory practice and good manufacturing practice regulations, 2) good laboratory practice toxicology studies, and 3) preparation of the investigational new drug package. Focus areas requiring support for clinical research included 1) clinical trial design and 2) correlative biomarker development. Respondents also called for a focus on resources—notably, access to pharmaceutical company and biotechnology partners as well as venture capital.

### Addressing the gap: the Accelerating Anticancer Agent Development and Validation–IOTN workshop

Based on identified priority areas, the CTTF collaborated with Accelerating Anticancer Agent Development and Validation (AAADV;https://aaadv.org/), a not-for-profit educational initiative of the FDA, academia, advocates, and industry that provides education and resources to help researchers accelerate development and delivery of cancer treatments to patients, to host a satellite workshop at the AAADV annual meeting. Speakers included key representatives of the FDA, NIH, venture capital, and pharma venture arms as well as academics who had successfully translated immuno-oncology platforms to clinical trials. The following key recommendations to bridge gaps in clinical development of immuno-oncology platforms emerged:

- **Discovery and product development:** The emphasis must include both scientific discovery and what is often considered more mundane product development but constitutes the bulk of the risk in getting from concept to a viable product. The latter includes a focus on manufacturability and scalability, attention to intellectual property, recognition of the critical importance of timelines, well-defined milestones tied to regulatory strategy, and recognition that although high-quality science is necessary for success, it is never sufficient: The engineering aspects of product development are what convert a discovery into a successful product.
- **Data integration:** It is critical to have succinct, complete, and comprehensive data packages supporting each key investment criterion. These packages include robust determination of the mechanism of action to structure activity relationships, target engagement, pharmacokinetics, toxicology and toxicokinetics, biomarker monitoring, and efficacy estimation. The Clinical and Translational Science Awards program approaches these needs with a strong leaning toward the clinical trial support functions listed earlier but often does not address early-stage development functions. Support structures akin to the Clinical and Translational Science Awards program but with comprehensive coverage of both early-stage and late-stage developmental needs are a must for successful clinical translation. Such a structure should also incorporate extensive bioinformatics, cheminformatics, and biostatistics components to ensure rigorous and consistent representation of all data packages, coupled with robust statistical analysis to ensure the validity of conclusions drawn.
- **Forum for academic-pharma partnerships:** Large multicenter phase 3 clinical studies will typically cost approximately $100 million (2023 rates). Investments of this magnitude will invariably require the participation of a large pharmaceutical company because these funding levels are outside the range of most other sources. Pharma participation in such product development is therefore practically inevitable. At the same time, seeking to balance risk with cost, pharma companies are willing to consider investing or partnering at all stages of development when the data are compelling. Unlike grant programs, however, which evaluate projects based on a single written document, pharma investments involve multiple rounds of evaluations and require considerable give and take between the pharma entity and the originating organization. Creating forums for increased interactions between academia and pharma is therefore a critical need. The need for such interaction takes on a particularly critical urgency in the setting of the IOTN, where an accelerated pace of discovery has led to many candidate therapeutic approaches becoming available. It is more important than ever that interactions with pharma are also accelerated to prevent this step from becoming the bottleneck. The next phase of the IOTN must therefore include forums for these discussions. Beyond matchmaking and facilitating interactions, the next phase of IOTN must include grant programs that require a pharma partner that contributes intellectually, thus ensuring that translational research has pharma development in mind from the onset and paves the way to eligibility for the orders-of-magnitude greater investment required of pharma companies to support product development.
- **Intellectual property support:** The need for higher-quality support of intellectual property development efforts emerges as a consistent theme. Although all universities and most stand-alone cancer centers have dedicated technology-transfer offices and are adept at filing invention disclosures and rapidly filing patent applications, skill in developing an intellectual property portfolio around a central invention theme and creating a defensible portfolio that provides freedom to operate are much rarer commodities, with few successful instances evident. Yet, secure intellectual property is a necessary component of IOTN discoveries that may be successfully translated to the clinic. Provision of comprehensive intellectual property portfolio development resources is therefore a necessity for the success of IOTN discoveries.
- **Networking opportunities:** It is also evident that multiple professions must work together to effect successful translation of discoveries to products. Basic scientists, engineers, clinicians, lawyers, management professionals, and pharmaceutical development specialists all need to come together to find the right combination for a given development effort. A daunting task under any circumstances, this challenge is further exacerbated by the need to accelerate progress to meet the goals of the Moonshot program. The IOTN needs to provide increased opportunities for direct networking, bringing together all necessary skill sets for successful development to take place.

### Conclusions and recommendations for the next phase of the IOTN to achieve a decade of progress in 5 years

Specific recommendations by the CTTF include novel funding mechanisms, new workshops, creation of an IOTN Foundation, and creation of a navigator resource to direct researchers to numerous available resources to enhance translational research. The CTTF recommends the creation of multiple requests for applications, including those that continue basic and translational research but, more importantly, requests for applications that drive toward a clinical trial endpoint. The CTTF also recommends structural elements in programs that support continuing refinement of the immunotherapy clinical trial landscape.

#### Novel funding mechanisms

First, the CTTF recommends creation of requests for applications with a phased or staged funding mechanism consisting of 3 phases: the first phase establishing proof of concept in animal models, the second phase supporting planning and preparation for a clinical trial, and the third phase supporting the trial itself. Such mechanisms would be nondilutive and therefore highly attractive to parallel venture and/or pharma investment. A template that could be followed is the R61/33 mechanism currently used in some of the NIH Rapid Acceleration of Diagnostics programs to rapidly develop diagnostics for COVID-19. Such mechanisms would include a mandatory regulatory agency interaction component (eg, pre–investigational new drug meeting), with the intention of accelerating development activities.

Second, the CTTF recommends funding mechanisms that support independent development of biomarkers and surrogate markers separately from a therapeutic approach. This mechanism is critical because proposals to develop surrogate markers that are not paired with a novel therapy generally score poorly at study sections because of a perceived lack of significance, even when those biomarkers have strong potential to guide the use of existing and future therapies.

Third, the CTTF suggests a funding mechanism that requires a “development partner,” such as a venture capitalist or a pharmaceutical company. The value of such a mechanism includes built-in vetting of “project maturity” by the development partner and substantial incentives for investment by the development partner because the federal funds would not be dilutive.

Fourth, the CTTF recommends a funding mechanism dedicated to good manufacturing practice and good laboratory practice testing. The NCI Experimental Therapeutics program performs these activities at NCI facilities on behalf of outside investigators, following a competitive review process. Capacity is limited, however, and not immuno-oncology specific; therefore, additional mechanisms are needed. Small Business Innovation Research Phase II is a possibility, but applicants must compete with the full spectrum of projects that are proposed to that mechanism, and study sections tend to sideline rote manufacturing applications. Yet, this important step is often an insurmountable roadblock to development. Providing an R01-Good Manufacturing Practice Manufacturing Required–type mechanism would be a huge benefit.

Finally, when there is a specific focus on immuno-oncology clinical trials, the CTTF suggests an R01-type mechanism specifically for immuno-oncology, with a clinical trial required. This requirement is particularly important in the setting of unique dose-escalation strategies that apply to immuno-oncology, where, unlike conventional maximum tolerable dose approaches used for chemotherapeutics, a minimum efficacious dose approach is preferable. Novel trial designs would therefore relax traditional assumptions of monotonic relationships between adverse event severity and efficacy, recognize that immunotherapy-related adverse events could be chronic phenomena, and be designed with combination therapies incorporating independent temporal scales in mind. Such trials would also preferably use the “Network” component of IOTN to accelerate phase 1 to 2 trials.

#### Recommendations for workshops and new resources

The CTTF recommends organizing workshops such as the Vail clinical trials skill development workshop (https://vailworkshop.org/) but focused on phase 1 trials for immuno-oncology, particularly educating the community on approaches relevant to immuno-oncology development. The CTTF proposes workshops on intellectual property, technology transfer, and technology licensing as they apply to immuno-oncology. This need is driven by the wide range of experience with intellectual property and technology transfer of immuno-oncology researchers as well as the nuances of drug development in the immuno-oncology space.

The CTTF also recommends creating a new resource, an IOTN Foundation, which would be modeled after similar foundations, such as the GOG Foundation (33) and NSABP Foundation (34). The formation of a 501(c)(3) nonprofit IOTN Foundation would enable investigators to support their clinical trial efforts in partnership with industry sponsors. The goals of the IOTN Foundation would be to 1) support trials that fall outside the traditional NCI funding stream, 2) create a point of contact for industry representatives to link to IOTN investigators, and 3) rapidly translate preclinical research findings into executable phase 1 and phase 1/2 trials. The IOTN Foundation would provide investigators with core infrastructure needs, such as legal support, financial management, protocol editing, trial monitoring and auditing support, data coordination, and biostatistical support. The IOTN Foundation would also establish a network of researchers and associated clinical sites willing to enroll patients in trials. As the IOTN Foundation becomes financially self-sufficient, we would expect to establish further enabling resources, such as specific cancer subcommittees, a tissue bank, educational activities, and support for early-stage investigator pilot projects (34,35).

As another new resource, the CTTF recommends formation of a navigator resource as a 1-stop shop that lists all available resources and pathways through the NIH, industry, and other resources available for immuno-oncology translation. Such a road map would be a huge benefit to all researchers, providing easy access to all relevant resources in 1 easy-to-navigate location. In conclusion, the first 5 years of the IOTN program have been highly productive in generating new insights into mechanisms of cancer as well as barriers to translation. We defined key areas to overcome these barriers and proposed new funding mechanisms and other resources to increase translation and reduce cancer deaths.

## Supplementary Material

djad151_Supplementary_Data

## Data Availability

No data was analyzed for this commentary.
