# Supplementary material for: The Cancer Moonshot Immuno-Oncology Translational Network at 5: accelerating cancer immunotherapies
Source: J Natl Cancer Inst. 2023 Aug 12;115(11):1262–70. doi: 10.1093/jnci/djad151 (PMC10637038; doi:10.1093/jnci/djad151)
Supplement: djad151_Supplementary_Data [file djad151_supplementary_data.pdf]

**Supplementary Materials**
Annapragada et al. Cancer Moonshot Immuno-Oncology Translational Network (IOTN)

**Supplementary Table 1: Members of the IOTN Clinical Trials Task Force (CTTF)**

| Member Name | Affiliation | CTTF designation |
|---|---|---|
| **Kunle Odunsi** | IOTN/ University of Chicago Medicine | CTTF Chair |
| **Andrew Sikora** | IOTN/ MD Anderson Cancer Center | CTTF Co-Chair |
| **Ananth Annapragada** | IOTN/ Baylor College of Medicine | Member |
| **Michael Demetriou** | IOTN/ University of California- Irvine | Member |
| **Lawrence Fong** | IOTN/ University of California- San Francisco | Member |
| **Jinming Gao** | IOTN/ University of Texas Southwestern | Member |
| **Alan Hutson** | IOTN/ Roswell Park Comprehensive Cancer Center | Member |
| **Donald Kufe** | IOTN/ Dana Farber Cancer Institute | Member |
| **Song Liu** | IOTN/ Roswell Park Comprehensive Cancer Center | Member |
| **Zachary Morris** | IOTN/ University of Wisconsin-Madison | Member |
| **Eduardo Vilar-Sanchez** | IOTN/ MD Anderson Cancer Center | Member |
| **Kasia Bourcier** | NIH/ NCI | Member |
| **Marc Ernstoff** | NIH/NCI | Member |
| **Lori Henderson** | NIH/ NCI | Member |
| **Kevin Howcroft** | NIH/ NCI | Member |
| **Lillian Kuo** | NIH/ NCI | Member |
| **Shizuko Sei** | NIH/NCI | Member |
| **Elad Sharon** | NIH/NCI | Member |
| **Robert Shoemaker** | NIH/NCI | Member |
| **Minkyung Song** | NIH/NCI | Member |
| **Anthony Welch** | NIH/ NCI | Member |
| **Jason Yovandich** | NIH/NCI | Member |
